# Performance of different CT enhancement quantification methods as predictors of pancreatic cancer recurrence after upfront surgery

**DOI:** 10.1038/s41598-024-70441-3

**Published:** 2024-08-26

**Authors:** Sherif A. Mohamed, Alina Barlemann, Verena Steinle, Tobias Nonnenmacher, Michelle Güttlein, Thilo Hackert, Martin Loos, Matthias M. Gaida, Hans-Ulrich Kauczor, Miriam Klauss, Philipp Mayer

**Affiliations:** 1 Clinic for Diagnostic and Interventional Radiology, Heidelberg University Hospital, Im Neuenheimer Feld 420, 69120 Heidelberg, Germany; 2 Department of Neuroradiology, Medical Faculty Mannheim, Heidelberg University, Theodor-Kutzer-Ufer 1-3, 68167 Mannheim, Germany; 3 Department of General, Visceral and Thoracic Surgery, University Hospital Hamburg-Eppendorf, Hamburg, Germany; 4 Clinic of General, Visceral, and Transplantation Surgery, Heidelberg University Hospital, Heidelberg, Germany; 5 Institute of Pathology, University Medical Center Mainz, JGU-Mainz, Mainz, Germany; 6 TRON, Translational Oncology at the University Medical Center, JGU-Mainz, Mainz, Germany

**Keywords:** Pancreatic cancer, Pancreatic ductal adenocarcinoma, Computed tomography, Enhancement, Recurrence, Upfront surgery, Cancer imaging, Pancreatic cancer

## Abstract

The prognosis of pancreatic cancer (PDAC) after tumor resection remains poor, mostly due to a high but variable risk of recurrence. A promising tool for improved prognostication is the quantification of CT tumor enhancement. For this, various enhancement formulas have been used in previous studies. However, a systematic comparison of these formulas is lacking. In the present study, we applied twenty-three previously published CT enhancement formulas to our cohort of 92 PDAC patients who underwent upfront surgery. We identified seven formulas that could reliably predict tumor recurrence. Using these formulas, weak tumor enhancement was associated with tumor recurrence at one and two years after surgery (p ≤ 0.030). Enhancement was inversely associated with adverse clinicopathological features. Low enhancement values were predictive of a high recurrence risk (Hazard Ratio ≥ 1.659, p ≤ 0.028, Cox regression) and a short time to recurrence (TTR) (p ≤ 0.027, log-rank test). Some formulas were independent predictors of TTR in multivariate models. Strikingly, almost all of the best-performing formulas measure solely tumor tissue, suggesting that normalization to non-tumor structures might be unnecessary. Among the top performers were also the absolute arterial/portal venous tumor attenuation values. These can be easily implemented in clinical practice for better recurrence prediction, thus potentially improving patient management.

## Introduction

Pancreatic ductal adenocarcinoma (PDAC) constitutes the most common malignant tumor of the pancreas and is the third to fourth-leading cause of cancer-related mortality in Western countries^1^. Even after radical tumor resection, 5-year overall survival rates of only about 20% can be expected^2,3^, mostly due to high rates of early locoregional and distant recurrence^4^. On a patient level, time to recurrence (TTR) and overall survival (OS) after tumor resection are highly heterogeneous with some patients dying from early recurrence within a few months post surgery and other patients living well beyond 5 years without recurrence^5^.

Clinicopathological factors such as the Tumor-Node-Metastasis (TNM) stage according to the American Joint Committee of Cancer (AJCC) or the Union for International Cancer Control (UICC) are frequently used predictive factors of TTR and OS^6,7^. However, prediction of TTR and OS based on these factors can be imprecise^8^ and most clinicopathological prognosticators failed to predict tumor recurrence in a large retrospective study^5^.

Improved risk stratification of PDAC patients would be beneficial for patient management^9^. Multidetector computed tomography (MDCT) is the preferred imaging modality for pancreatic cancer according to major guidelines^10,11^. Therefore, numerous radiological studies focused on the utilization of quantitative CT features to improve PDAC prognostication^9^.

A simple, yet effective quantitative method is the measurement of CT enhancement which has been utilized for the characterization of PDAC for 50 years^12^. Various studies have been conducted on the prediction of clinical endpoints (e.g. TTR and OS) or adverse histopathological tumor features (e.g. histopathological grading and tumor cellularity) with promising results^13,14^. These studies used different measurement approaches for enhancement quantification. Some groups used simple absolute attenuation measurements of the tumor in the (late) arterial and venous phase^15^, or attenuation differences or quotients^16^. Others normalized the tumor enhancement to the aortic enhancement^13^ or calculated the tumor enhancement relative to the enhancement of the non-neoplastic pancreatic parenchyma^16^, sometimes at the tumor-pancreas interface^16^.

The aforementioned studies included only CT scans from one or two scanner model(s) and with one fixed scanning protocol^13–17^. Simple attenuation measurements of the pancreas without normalization, as performed in some studies^15^, are said to be affected by inter-scanner variability^18^. This might, in theory, negatively affect the transferability of these measurements to clinical routine^19^. Normalization of the enhancement to the aorta or non-neoplastic parenchyma could reduce the effect of differences in scanner hardware, contrast agent bolus, and reconstruction algorithm^19^. However, since many enhancement formulas have never been tested in independent datasets (Supplementary Table 1) and systematic comparisons are lacking, it is not known which formula or measurement is best for risk stratification of PDAC patients.

Therefore, the present exploratory PDAC study aimed to evaluate the different published enhancement measurements/ formulas for the prediction of TTR after upfront surgery, using CT scans from various scanner models.

## Results

### Patient characteristics

A total of 189 patients with preoperative three-phase abdominal CT examination, upfront pancreatic tumor resection, and final histopathological diagnosis of PDAC were retrospectively identified from our radiological database. Excluded were 2 patients with inadequate image quality, 22 patients where the exact tumor extension in CT was not clear ((partially) occult tumor and/or severe concomitant pancreatitis), and 73 recurrence-free patients with follow-up < 2 years. Subsequently, the data of 92 patients (46 female, 46 male) were analyzed. A flowchart of the study population is presented in Supplementary Fig. 1.The radiological median maximum lesion diameter was 28 (interquartile range (IQR) 20–36) mm.

Information on performed pancreatic resections is presented in the Supplementary Materials (page 2).

Additional clinicopathological characteristics of the study population are shown in Table 1.

**Table 1:** Demographic & clinical data and CT enhancement values in patients without and with tumor recurrence within 1 year.

		Patients without recurrence within 1 year (n = 41)	Patients with recurrence within 1 year (n = 51)	p-value
Clinical and pathological data				
Sex	Female	24	22	0.144
	Male	17	29	
Age, median (IQR) [years]		68.5 (57.7–74.5)	66.3 (59.7–72.1)	0.900
T stage	1	9	8	0.186
	2	28	29	
	3	4	13	
	4	0	1	
M stage	0	16	8	**0.035**
	1	11	16	
	2	14	27	
M stage	0	40	49	0.692
	1	1	2	
Grading	1	1	0	**0.003**
	2	32	24	
	3	8	27	
CA19-9^21^	< 100 U/mL	25	18	**0.015**
	≥ 100 U/mL	16	33	
Enhancement values, median (IQR)				
Measurement **(1)** ${Tu}_{art}$		61.0 (54.8–75.8) HU	52.5 (41.7–65.1) HU	**0.002**
Measurement **(2)** ${Tu}_{ven}$		73.1 (61.8–86.7) HU	63.2 (56.4–85.7) HU	**0.020**
Formula **(3)** ${Tu}_{art}-{Tu}_{nc}$		29.1 (21.8–44.3) HU	20.0 (13.1–31.0) HU	**0.005**
Formula **(4)** ${Tu}_{ven}-{Tu}_{nc}$		43.1 (32.1–60.1) HU	38.6 (22.7–52.8) HU	**0.030**
Formula **(6)** $\frac{{Tu}_{art}}{{Tu}_{nc}}$		1.98 (1.58–2.59)	1.64 (1.39–2.06)	**0.011**
Formula **(8)** ${Tu}_{art}-\frac{{Tu}_{nc}}{{Tu}_{nc}}$		0.98 (0.58–1.59)	0.64 (0.39–1.06)	**0.011**
Formula **(10)** $\frac{{Tu}_{art}}{{Aorta}_{art}}$		0.22 (0.18–0.30)	0.18 (0.14–0.23)	**0.002**
Formula **(11)** $\frac{{Tu}_{ven}}{{Aorta}_{ven}}$		0.58 (0.46–0.67)	0.50 (0.38–0.60)	**0.013**
Formula **(13)** $\frac{{Tu}_{art}-{Tu}_{nc}}{{Aorta}_{art}-{Aorta}_{nc}}$		0.12 (0.10–0.17)	0.08 (0.05–0.13)	**0.001**
Formula **(14)** $\frac{{Tu}_{ven}-{Tu}_{nc}}{{Aorta}_{ven}-{Aorta}_{nc}}$		0.47 (0.33–0.58)	0.40 (0.21–0.51)	**0.012**

*art* late arterial, *CA19-9* carbohydrate antigen 19–9, *HU* Hounsfield units, *IQR* interquartile range. *M* metastasis, *N* nodal, *nc* non-contrast, *ven* portal venous, *T* tumor, *Tu* tumor.

Significant values are in bold.

### Tumor recurrence

The median follow-up time for all patients was 671 (IQR 346–1257) days.

77 patients showed tumor recurrence after 50–3085 days, median 305 (IQR 164–466) days. 20 patients had a locoregional recurrence. 40 patients had distant metastasis(es) (17 liver, 5 peritoneum, 6 lung, 2 distant lymph nodes, 10 multiple sites). 17 patients had both locoregional and distant recurrence.

15 patients showed no signs of tumor recurrence after a follow-up time of 843–3501 days, median 1660 (IQR 1085–2521) days.

### Placement of regions of interest and extraction of attenuation values

**Figure 1:**
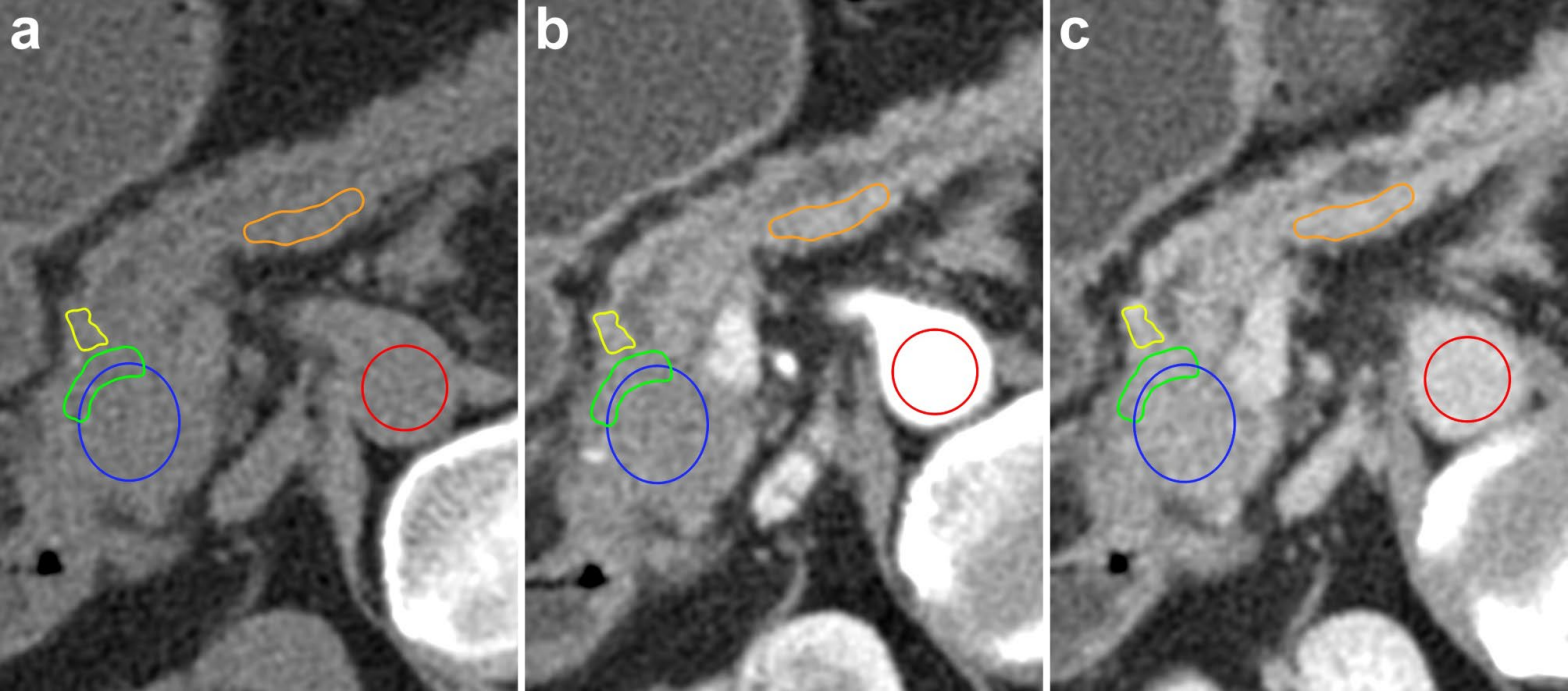
Placement of regions of interest (ROIs). Shown are the ROIs in a patient with a PDAC in the pancreatic head in the (**a**) non-contrast, (**b**) late arterial, and (**c**) portal venous phase; CT acquired in an oblique, 30°, right-sided down position^20^. Blue ROI *tumor*: oval/round, centered in the middle of the tumor, with a margin of at least 2 mm to the border of the tumor. Green ROI *tumor periphery*: freehand, approximately 5 mm thick, peripheral tumor part. Yellow ROI *upstream parenchyma—border to tumor*: freehand, upstream parenchyma directly adjacent to the tumor. Orange ROI *upstream parenchyma—best measurable:* oval or freehand, best measurable area of upstream non-neoplastic tissue. Red ROI *aorta*: oval/round, in the aortic lumen, diameter ~ 15 mm.

Attenuation values for every CT phase (non-contrast, late arterial, portal venous) were extracted from the following regions of interest (ROIs): tumor (covering a relevant proportion of the tumor area, with a margin of at least 2 mm to the border of the tumor, n = 92); tumor periphery (n = 92); upstream parenchyma (best measurable area, n = 72); upstream parenchyma (border to the tumor, n = 72); downstream parenchyma (best measurable area, n = 45); aorta (n = 92). For more details on ROI placement see the Materials and Methods section, the Supplementary materials (page 4) and Fig. 1.

### Extraction of CT enhancement measurements/formulas from previous studies

Using a PubMed^®^ MEDLINE and PubMed Central^®^ search, sixteen PDAC studies were identified that used CT enhancement measurements of PDAC for prediction of patient prognosis or of prognostically relevant histopathological tumor features between 01/2009 and 01/2024.

The following enhancement measurements/ formulas were extracted from the identified studies:

(A) Enhancement of tumor without normalization to non-tumor structures:

1. $${Tu}_{art}$$
2. $${Tu}_{ven}$$
3. $${Tu}_{art}-{Tu}_{nc}$$
4. $${Tu}_{ven}-{Tu}_{nc}$$
5. $${Tu}_{ven}-{Tu}_{art}$$
6. $$\frac{{Tu}_{art}}{{Tu}_{nc}}$$
7. $$\frac{{Tu}_{ven}}{{Tu}_{nc}}$$
8. $${Tu}_{art}-\frac{{Tu}_{nc}}{{Tu}_{nc}}$$
9. $${Tu}_{ven}-\frac{{Tu}_{nc}}{{Tu}_{nc}}$$

(B) Enhancement of tumor normalized to aortic enhancement:

(10) $$\frac{{Tu}_{art}}{{Aorta}_{act}}$$
(11) $$\frac{{Tu}_{ven}}{{Aorta}_{ven}}$$
(12) $$\frac{{Tu}_{ven}-{Tu}_{art}}{{Aorta}_{art}-{Aorta}_{ven}}$$
(13) $$\frac{{Tu}_{art}-{Tu}_{nc}}{{Aorta}_{art}-{Aorta}_{nc}}$$
(14) $$\frac{{Tu}_{ven}-{Tu}_{nc}}{{Aorta}_{ven}-{Aorta}_{nc}}$$

(C) Enhancement of tumor in relation to non-neoplastic parenchyma:

(15) $${Upstream}_{art}-{Tu}_{art}$$
(16) $${Upstream}_{ven}-{Tu}_{ven}$$
(17) $$\frac{{Tu}_{ven}-{Tu}_{art}}{{Upstream}_{ven}-{Upstream}_{art}}$$
(18) $$\frac{{Tu}_{art}}{{Upstream}_{art}}$$
(19) $$\frac{{Tu}_{ven}-{Tu}_{nc}}{{Upstream}_{ven}-{Upstream}_{nc}}$$

(D) Enhancement of interface between tumor and non-neoplastic parenchyma:

(20) $${Upstream}_{border, art}-{Tu}_{periphery,art}$$
(21) $${Upstream}_{border, ven}-{Tu}_{periphery,ven}$$
(22) $$\frac{{Upstream}_{border,art}}{{Tu}_{periphery,art}}$$
(23) $$\frac{{Upstream}_{border,\text{ven}}}{{Tu}_{periphery,\text{ven}}}$$

For more details see Supplementary Materials (pages 5–7), including Supplementary Table 1.

### Comparison of attenuation values from regions on interest

Attenuation values from the ROIs are summarized in the Supplementary Materials (page 7), including Supplementary Table 2.

### Correlation of enhancement values from enhancement formulas with clinicopathological parameters

Correlations/ associations (p < 0.05) were observed between enhancement values from formulas (2), (4), (7), (9), (11), (14), (20), (22) with patient age, between values from formulas (2), (10), (11), (14) with grading, between values from formulas (1), (3), (5), (12), (13) and lymph-node metastasis(es), between formulas (1), (3), (6), (8), (12) and UICC stage, and between values from formulas (3), (6), (7), (8), (9), (13) and radiological tumor size (see Supplementary Materials (page 8) for a detailed analysis).

### Prediction of tumor recurrence by CT enhancement values and clinicopathological features

**Figure 2:**
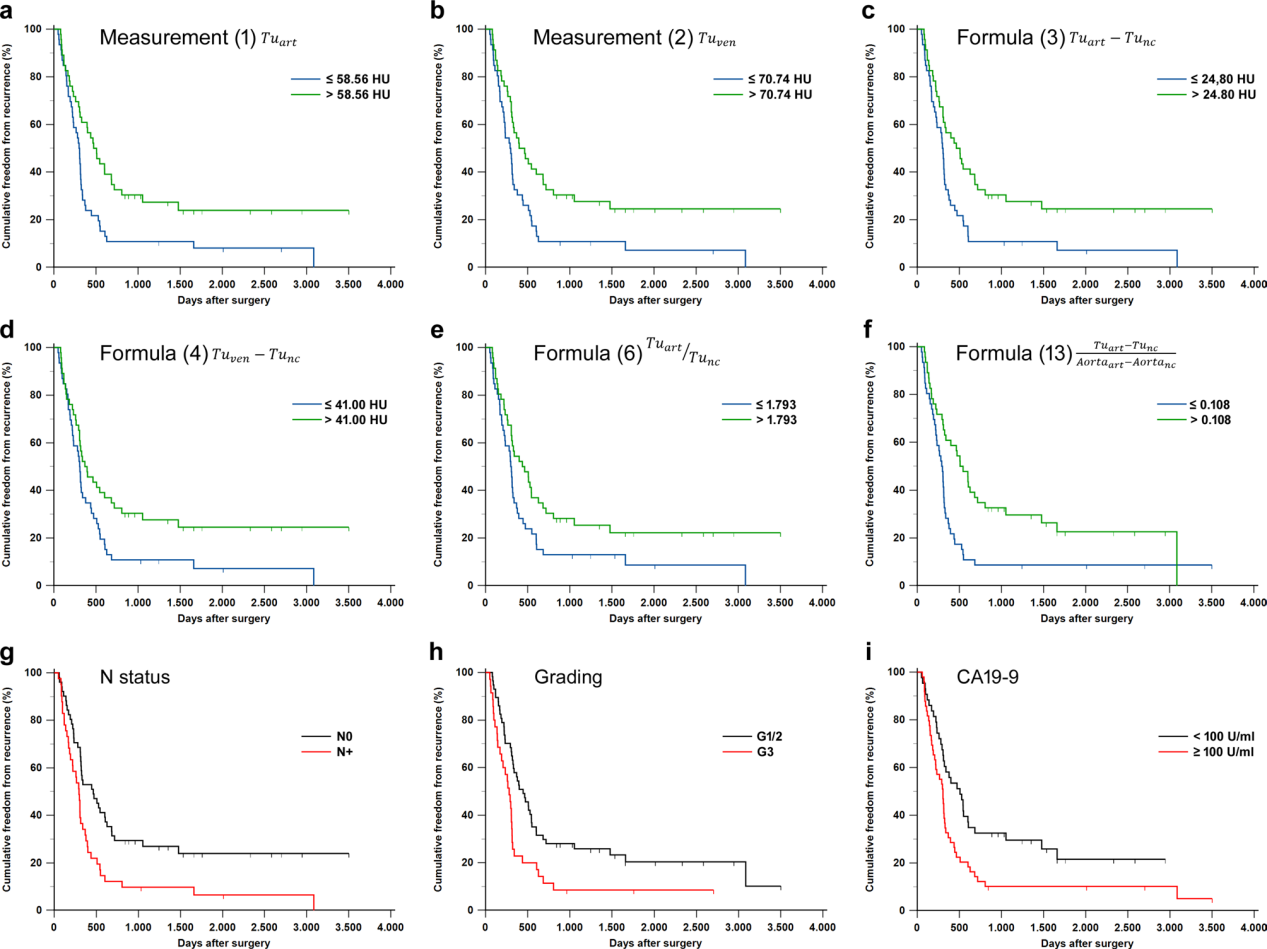
Kaplan Meier curves for cumulative freedom from recurrence (**a**–**f**) stratified by enhancement values from measurements/formulas (≤ median *versus* > median) associated with the lowest p—values (0.004 ≤ p ≤ 0.027, log-rank test), (**g**–**i**) and stratified by the clinicopathological variables nodal (N) status (N0 *versus* N +), histopathological grading (G1/2 *versus* G3), as well as serum carbohydrate antigen 19–9 (CA19-9) (< 100 U/ml *versus* ≥ 100 U/ml)^21^ (0.003 ≤ p ≤ 0.012). Censored patients are shown as tick marks. Please note that Kaplan Meier curves from formula 8 are not shown since they are identical to curves from formula 6. Additional abbreviations *art* late arterial, *nc* non-contrast, *Tu* tumor, *ven* portal venous.

According to univariate Kaplan–Meier analysis and log-rank test, TTR differences with p < 0.05 between patients with above and below median enhancement values were detected for formulas (1), (2), (3), (4), (6), (8), and (13). For these formulas, patients with low enhancement values had shorter TTR than patients with high enhancement values (p ≤ 0.027, n = 92). Kaplan–Meier curves for these formulas are presented in Fig. 2. The results from the Kaplan–Meier analysis and log-rank test for all enhancement formulas are summarized in Supplementary Table 3.

Among clinical and pathological variables, larger tumor size (≥ 28 mm), presence of lymph node metastasis(es), high histopathological grade (G3), and high serum carbohydrate antigen 19–9 (CA19-9) (≥ 100 U/ml)^21^ were associated with shorter TTR (p ≤ 0.026, log-rank test, n = 92) (Supplementary Table 4).

In univariate Cox regression analyses, above versus below median enhancement values from the same formulas ((1), (2), (3), (4), (6), (8), (13)) were associated with low recurrence risk (Hazard Ratio (HR) ≤ 0.603, p ≤ 0.028, n = 92). Formula (13) yielded the highest Harrel’s C-index (C = 0.595, 95% CI 0.541–0.650). A comparison of the lowest C-index from formula (5) (C = 0.502, 95% CI 0.444–0.560) with the C-index from formula (13) yielded a p-value of 0.009, while comparisons with the C-indices from formula (1), (2), and (3) (0.575 ≤ C ≤ 0.577) resulted in p-values ≥ 0.083.

The results from the univariate Cox regression analyses with enhancement measurement as predictor variables are presented in Table 2.

**Table 2:** Univariate Cox regression analyses: enhancement as a predictor for tumor recurrence.

Covariate		n	Hazard ratio (95% CI)	p-value	Harrel’s C (95% CI)
Measurement (1)${Tu}_{art}$	> median *versus* ≤ median	92	0.518 (0.328–0.819)	**0.005**	0.577 (0.521–0.633)
Measurement (2)${Tu}_{ven}$			0.528 (0.335–0.833)	**0.006**	0.576 (0.521- 0.632)
Formula (3)${Tu}_{art}-{Tu}_{nc}$			0.523 (0.331–0.827)	**0.006**	0.575 (0.519–0.631)
Formula (4)${Tu}_{ven}-{Tu}_{nc}$			0.592 (0.376–0.933)	**0.024**	0.553 (0.496–0.610)
Formula (5)${Tu}_{ven}-{Tu}_{art}$			1.009 (0.645–1.578)	0.970	0.502 (0.444–0.560)
Formula (6)$\frac{{Tu}_{art}}{{Tu}_{nc}}$			0.603 (0.383–0.948)	**0.028**	0.562 (0.506–0.618)
Formula (7)$\frac{{Tu}_{ven}}{{Tu}_{nc}}$			0.657 (0.417–1.033)	0.069	0.535 (0.478–0.593)
Formula (8)${Tu}_{art}-\frac{{Tu}_{nc}}{{Tu}_{nc}}$			0.603 (0.383–0.948)	**0.028**	0.562 (0.506–0.618)
Formula (9)${Tu}_{ven}-\frac{{Tu}_{nc}}{{Tu}_{nc}}$			0.657 (0.417–1.033)	0.069	0.535 (0.478–0.593)
Formula (10)$\frac{{Tu}_{art}}{{Aorta}_{art}}$			0.648 (0.412–1.019)	0.060	0.556 (0.499–0.614)
Formula (11)$\frac{{Tu}_{ven}}{{Aorta}_{ven}}$			0.754 (0.481–1.180)	0.217	0.532 (0.475–0.590)
Formula (12)$\frac{{Tu}_{ven}-{Tu}_{art}}{{Aorta}_{art}-{Aorta}_{ven}}$			0.919 (0.586–1.442)	0.714	0.521 (0.463–0.579)
Formula (13)$\frac{{Tu}_{art}-{Tu}_{nc}}{{Aorta}_{art}-{Aorta}_{nc}}$			0.487 (0.307–0.772)	**0.002**	0.595 (0.541–0.650)
Formula (14)$\frac{{Tu}_{ven}-{Tu}_{nc}}{{Aorta}_{ven}-{Aorta}_{nc}}$			0.708 (0.451–1.110)	0.708	0.535 (0.477–0.592)
Formula (15)${Upstream}_{art}-{Tu}_{art}$		72	0.814 (0.489–1.353)	0.427	0.522 (0.457–0.587)
Formula (16)${Upstream}_{ven}-{Tu}_{ven}$			1.154 (0.691–1.927)	0.585	0.506 (0.440–0.571)
Formula (17)$\frac{{Tu}_{ven}-{Tu}_{art}}{{Upstream}_{ven}-{Upstream}_{art}}$			1.292 (0.772–2.161)	0.329	0.525 (0.460–0.590)
Formula (18)$\frac{{Tu}_{art}}{{Upstream}_{art}}$			1.384 (0.828–2.313)	0.216	0.555 (0.492–0.618)
Formula (19)$\frac{{Tu}_{ven}-{Tu}_{nc}}{{Upstream}_{ven}-{Upstream}_{nc}}$			0.677 (0.407–1.128)	0.134	0.549 (0.486–0.613)
Formula (20) ${Upstream}_{border, art}-{Tu}_{periphery,art}$			0.776 (0.464–1.300)	0.332	0.525 (0.460–0.589)
Formula (21) ${Upstream}_{border, ven}-{Tu}_{periphery,ven}$			1.000 (0.600–1.667)	0.999	0.510 (0.445–0.574)
Formula (22)$\frac{{Upstream}_{border,art}}{{Tu}_{periphery,art}}$			1.100 (0.659–1.836)	0.715	0.525 (0.460–0.590)
Formula (23)$\frac{{Upstream}_{border,ven}}{{Tu}_{periphery,ven}}$			1.079 (0.647–1.799)	0.772	0.521 (0.457–0.586)

*art* late arterial, *CI* confidence interval, *nc* non-contrast, *ven* portal venous, *T* tumor, *Tu* tumor *Upstream* upstream parenchyma.

Significant values are in bold.

Among the clinicopathological variables, large radiological tumor size (≥ 28 mm), presence of lymph node metastasis(es) (N), high histopathological grading (G = 3), and high serum CA19-9 (≥ 100 U/ml)^21^ were associated with high tumor recurrence risk in univariate Cox regression analyses (HR ≥ 1.682, p ≤ 0.028, n = 92, Supplementary Table 5). High grading (G = 3) was associated with the highest Harrel’s C-index (C = 0.586, 95% CI 0.534–0.639); comparison with the lowest clinicopathological C-index from serum carcinoembryonic antigen (CEA) (C = 0.519, 95%CI 0.465–0.573) yielded a p-value of 0.075.

Multivariate Cox proportional-hazards regression analyses were performed. First, a clinicopathological model was built with the following potential predictor variables (FORWARD method): T stage (≥ 3 versus ≤ 3), N status (N + versus N0), histopathological grading (3 versus ≤ 2), and serum CA19-9 (≥ 100 versus < 100 U/ml)^21^. The variables N status and grading (p ≤ 0.004) were included in the final model (p < 0.001) which had a Harrel’s C-index of 0.628 (95% CI 0.577–0.680, n = 92). In a multivariate clinical model with the ENTER method, N status and grading (p ≤ 0.027) remained significant, and T stage and CA19-9 were not significant (p ≥ 0.138). In a second step, combined models were built which, in addition to the clinical variables, included one of the four enhancement formulas/ measurements, each, that were associated with the highest C-indices and lowest p-values in univariate Cox regression analyses (formulas (1), (2), (3), and (13), C ≥ 0.575, p ≤ 0.006). In the models with measurements (1) (late arterial tumor attenuation) or (2) (portal venous tumor enhancement), these enhancement parameters were found not to contribute to the prediction of TTR and were not included in the models (FORWARD method), or included in the models (ENTER) but associated with p ≥ 0.056. The other combined FORWARD models included the respective enhancement formula ((3) or (13)) (p = 0.029; and p = 0.043) in addition to the clinicopathological variables and yielded slightly higher Harrel’s C-indices than the clinical model (C = 0.651, 95% CI 0.592–0.709; and C = 0.657, 95% CI 0.606–0.707; n = 92). In the combined ENTER models, only formula (3) was a significant predictor (p = 0.030) while formula (13) was associated with a p-value of 0.128. Comparisons of the highest C-index from a single clinicopathological variable (grading) with C-indices from the combined FORWARD models (incorporating formulas (3) or (13)) yielded p-values < 0.05 (0.012 ≤ p ≤ 0.029), but not with the C-index from the multivariate clinicopathological model (p = 0.248). Third, in preoperative multivariate models incorporating only the preoperatively available parameters CA 19–9, radiological tumor size, and enhancement measurements, the formulas/ measurements (1), (2), (3), and (13) were independent predictors of TTR (p ≤ 0.024, n = 92). The results from the multivariate Cox regression analyses are summarized in Supplementary Tables 6–9.

**Figure 3:**
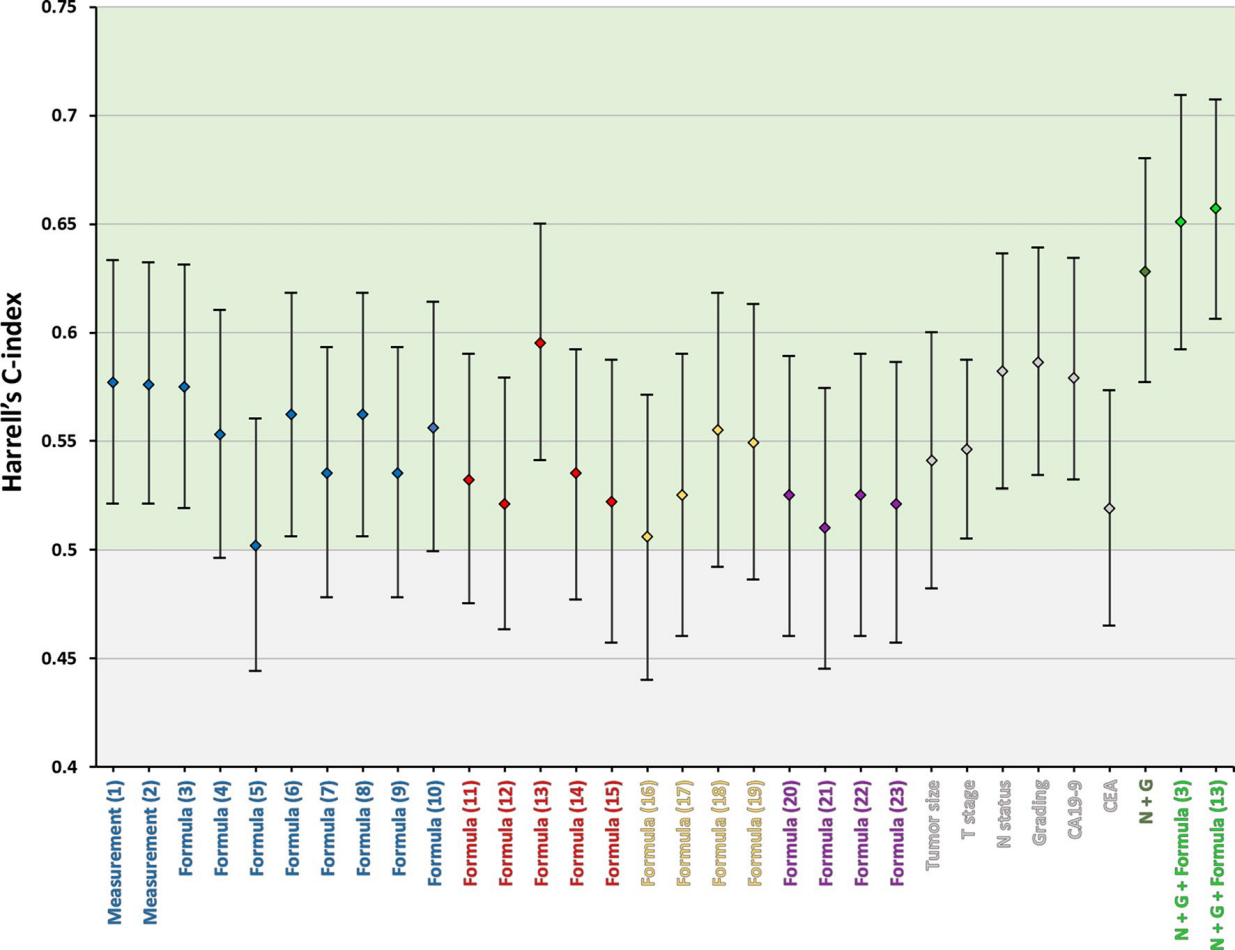
Harrell’s C-indices (diamonds) with 95% CI (whiskers) from Cox regression analyses for prediction of time to tumor recurrence (TTR). C-indices (univariate) are shown for enhancement formulas measuring solely tumor tissue (blue), measuring tumor tissue with normalization to the aorta (red), measuring tumor tissue in relation to non-neoplastic pancreatic parenchyma (gold), and measuring the interface enhancement (purple), median enhancement values as cut-off, each. Among all enhancement formulas, formula (13) yielded the highest C-index (0.595, 95% CI 0.541—0.650). For comparison, C-indices (univariate) from clinicopathological variables (grey) are shown for tumor size (≥ 28 mm *versus* < 28 mm), tumor (T) stage (T ≥ 3 *versus* T ≤ 2), nodal (N) status (N + *versus* N0), histopathological grading (G = 3 *versus* G ≤ 2), carbohydrate antigen 19-9 (CA19-9) (≥ 100 U/ml versus < 100 U/ml)^21^, and carcinoembryonic antigen (CEA) (≥ 2.5 ng/mL *versus* < 2.5 ng/mL)^22^. Among clinicopathological variables, grading yielded the highest C-index (0.586, 95% CI 0.534-0.639). A multivariate clinicopathological model (FORWARD) incorporating both N status and grading as predictors of TTR was associated with a C-index of 0.628 (95% CI 0.577—0.680, dark green). Multivariate combined models (FORWARD) in which both clinicopathological variables (N status & grading) and enhancement values from formula (3) or (13) were predictors of TTR yielded slightly higher C-indices (0.651, 95% CI 0.592-0.709; and 0.657, 95% CI 0.606-0.707; light green).

Harrel’s C-indices with 95% CIs from Cox regression analyses are shown in Fig. 3.

Presence of tumor recurrence at both 1 year and 2 years after surgery was associated with lower enhancement values from formulas (1), (2), (3), (4), (6), (8), (10), (13), (14) (p ≤ 0.039, n = 92). In addition, patients with tumor recurrence at 1 year had lower enhancement values from formula (11) (p = 0.013, n = 92) than patients without recurrence.

A comparison of clinicopathological parameters and enhancement parameters (with p < 0.05) between patients without and with tumor recurrence at 1 year is presented in Table 1.

In the receiver operating characteristic (ROC) curve analysis, among all enhancement measurements, the formulas (1), (3), (10), and (13) yielded the highest Area under the Curve (AUC) values and Youden indices (0.67 ≤ AUC ≤ 0.70; 0.339 ≤ Youden index ≤ 0.384; p ≤ 0.003; n = 92) for prediction of tumor recurrence at 1 year after surgery (Supplementary Table 10). Among the clinicopathological parameters, N status and histopathological grading were associated with the highest AUC values and Youden indices (0.63 ≤ AUC ≤ 0.67; 0.233 ≤ Youden index ≤ 0.334; p ≤ 0.016; n = 92). Differences in correspondent AUC values between formulas (1), (3), (10), & (13) and N status & histopathological grading were associated with p-values ≥ 0.288. Selected ROC curves are shown in Supplementary Fig. 2.

## Discussion

The present exploratory study identifies seven CT enhancement formulas that could reliably predict tumor recurrence in PDAC after upfront surgery. According to these formulas ((1), (2), (3), (4), (6), (8), and (13)), locoregional or distant tumor recurrence at one and two years after surgery was associated with weak tumor enhancement. Enhancement values from some of these formulas were correlated with adverse clinicopathological features such as histopathological grading, the presence of lymph node metastases, and serum CA19-9. Low CT enhancement values from these formulas were predictive of short time to recurrence (TTR) in univariate Kaplan–Meier analyses and a high tumor recurrence risk in univariate Cox regression analyses. (1), (2), (3), and (13) were the best-performing formulas in univariate analysis, and out of these, (3) and (13) were independent predictors of TTR in a Cox regression model incorporating the clinicopathological features T stage, N status, histopathological grading, and CA 19–9.

Strikingly, six out of the seven best-performing formulas measure solely tumor tissue without any normalization to non-tumor structures. However, the formula with the best predictive ability for tumor recurrence was formula (13) from a study by Torphy et al.^13^. The formula normalizes the tumor enhancement to the aortic enhancement in the arterial phase. In the original study by Torphy et al., the formula was also used to predict TTR as in the present study. However, in their study, this formula was outperformed by the equivalent formula for the portal venous phase^13^.

Notably, in our study, simple absolute attenuation measurements of the tumor in the late arterial (1) and portal venous phase (2) were also among the best prognostic factors on our heterogeneous CT dataset. This could mean that normalization of PDAC attenuation values to non-tumor structures for minimizing the influence of technical scanner and protocol variations^6^ is not strictly necessary.

Other formulas with good predictive ability in the present study ((3), (4), (8)) were also good prognosticators after tumor resection in previous studies^23,24^.

Various studies investigated the correlation between PDAC enhancement in CT and the presence and degree of prognostically relevant histopathological tumor features. A prognostically unfavorable weak PDAC enhancement was associated with the negative prognostic factors high tumor cellularity, high histopathological grading, and tumor necrosis^14,25–27^. However, the relationships between other histopathological features, CT enhancement, and clinical endpoints are more complex. Low late arterial tumor enhancement which was a negative prognosticator in the present and previous studies^23,28^ was associated with the beneficial prognostic markers low microvessel density and high content of fibrotic stroma^23,25,29^. Abundant stroma, though, was also linked to high portal venous tumor enhancement (positive prognosticator)^13^.

A relevant proportion of enhancement formulas analyzed in the present study (formulas (15)-(23)) incorporate both tumor enhancement and enhancement of the non-neoplastic parenchyma. A landmark renal cell carcinoma study where enhancement normalization to “normal” renal parenchyma (and the aorta) improved tumor characterization is often mentioned as a rationale for tumor enhancement normalization^19^. In the pancreas of PDAC patients, however, the non-neoplastic parenchyma is by no means “normal”. It is characterized by varying degrees of chronic inflammation and fibrosis both of which are independently associated with a poor prognosis^30^. In the present study, formulas evaluating the enhancement of the (whole) tumor to the non-neoplastic parenchyma (formulas (15)-(19)) yielded no relevant prognostic information. In line with this, in a study by Vyas et al., the absolute tumor attenuation (1), but not the tumor attenuation normalized to the non-neoplastic parenchyma in the late arterial phase (18) was predictive of survival in PDAC patients after pancreaticoduodenectomy^28^. One major limitation of most studies incorporating the non-neoplastic pancreatic parenchyma into their enhancement formula is the lack of differentiation between or pooling of upstream and downstream parenchyma^6,16,17,28,31–33^. In the present and a previous study, downstream parenchyma behaved similarly to the healthy pancreas with an enhancement peak at the late arterial phase^34^. This early peak is usually absent in the upstream parenchyma, possibly due to a higher degree of fibrous parenchymal replacement^34^. Therefore, in the present study, we avoided pooling upstream and downstream parenchyma but performed all analyses of the non-neoplastic parenchyma using solely upstream parenchyma (downstream parenchyma was available in fewer patients).

In our study, attenuation values of the non-neoplastic parenchyma were not age-dependent which is in contrast to a study by Itoh et al.^35^. We did, however, observe higher tumor enhancement values in above median age patients ((2), (4), (7), (11), (14), (18)) although the biological basis of this finding is not clear.

Some of the analyses in our study put a particular focus on the enhancement of the peripheral tumor parts or the interface of tumor and non-neoplastic parenchyma (formulas (20) -(23)). Biologically, the tumor periphery differs from the tumor center in terms of MVD, stroma content, and cellular density, all of which potentially influence enhancement^36,37^. In a few previous studies, high attenuation differences between the peripheral tumor parts and non-neoplastic parenchyma (“high delta”) were associated with adverse histopathological features (e.g. lymphovascular invasion) and poor clinical outcomes (e.g. high risk of recurrence)^16,32,33^. In our study, “high delta” tumors had no elevated risk of recurrence compared to “low delta” tumors which might be explainable by a potentially high variability in interpretation of the tumor margin^38^.

The present study only included PDAC patients after upfront surgery to generate a clinically more homogeneous patient dataset. As PDAC is increasingly seen as systemic disease, irrespectively of the initial stage, there currently is a paradigm shift in the management of initially resectable PDAC towards neoadjuvant therapy (NAT) which might improve TTR and OS in some patients^39^. Future radiology (enhancement) studies could help to identify patients with initially resectable PDAC that could profit from NAT.

In recent years, the proportion of radiology PDAC studies using Radiomics and Artificial Intelligence (AI) for postoperative prognosis estimation has been increasing. They often have a multicentric design which facilitates the inclusion of larger patient numbers^40,41^. The majority of these studies on PDAC rely on manual three-dimensional image segmentations to enable imaging feature extraction^41^. Some groups, however, recently managed to develop reliable automated (deep) segmentation and prognostic models which increases scalability and makes near-term translation into routine radiological practice more realistic^40^. Similar to the enhancement formula studies, several Radiomics/AI models also include information from the non-neoplastic parenchyma as well as clinical parameters for improved prognosis prediction^41^. Compared to the best-performing enhancement formulas, the prognostic capability of most Radiomics/AI models is still in the same range but is continuously improving^40,41^.

As shown in the present and previous studies, PDAC has a high but variable risk of recurrence after surgical tumor resection^5,8^. Insufficient information on the risk of recurrence can lead to patient anxiety and manifest PDAC recurrence is associated with a particularly high deterioration in health-related quality of life^42^. On a patient level, prognostic imaging markers such as the enhancement formulas from the present study and Radiomics/AI models could be a step in the direction toward more individualized PDAC surveillance programs after curative resection and more personalized treatment approaches to PDAC^43^.

Most guidelines, such as the National Comprehensive Cancer Network guideline, allow some flexibility regarding the frequency of follow-up CT scans (e.g. every 3–6 months for two years)^10^ and patients with a high recurrence risk (determined from risk assessments such as in the present study) could possibly profit from more frequent imaging (every 3 months). However, more data on the clinical benefit and cost-effectiveness of detection of recurrence at an early stage are desirable. Some older studies reported that routine postoperative imaging is not cost-effective as it might not have a significant impact on survival^44^, especially in patients with a poor performance status that are not fit for treatment of their recurrence^45^. Yet, there are more positive studies on imaging surveillance, such as a nationwide Dutch cohort study which inferred that postoperative surveillance with CA 19–9 and radiologic imaging has the potential to improve survival^46^. An ongoing international randomized controlled trial will provide further high-quality evidence on the clinical benefit and cost-effectiveness of a recurrence-focused surveillance (CA 19–9 & CT every three months) versus non-standardized surveillance (CA 19–9 & imaging only in case of symptoms)^47^.

There are some limitations to our study. First, we used TTR as the sole time-to-event endpoint since many patients were still alive at the end of follow-up. Second, the transferability of the findings from a single-center study might be limited. We did, however, include many external CT scans which added diversity to our CT dataset. Third, we did not perform image normalization prior to enhancement analyses because we wanted to keep the analyses technically simple and easy to repeat. Although we detected no or weak correlations between CT acquisition/ reconstruction parameters and enhancement measurements as well as TTR values, we cannot exclude that the technical variability had an influence on our results. Forth, the sample size of our study was moderate. Future larger (prospective) multi-center studies are desirable for an improved power of statistical analyses. Fifth, as this is an exploratory study and we wanted to avoid a high rate of false-negative findings, no alpha adjustment was done^48,49^. However, with this approach, one must be aware of an increased chance of false-positive findings in our studies. Thus, the positive findings from our exploratory study should be tested in future confirmatory studies.

In summary, our study identifies several CT enhancement formulas that have a prognostic ability to predict tumor recurrence in PDAC patients after upfront resection. Almost all of the best-performing formulas measure solely tumor tissue without any normalization to non-tumor structures. Among these top performers were the absolute tumor attenuation values in the late arterial and portal venous phase. The tumor attenuation can be easily measured on routine preoperative CTs and thus is more readily available than histopathological prognostic markers such as grading and lymph node metastasis which are difficult to determine preoperatively. Improved prediction of tumor recurrence could be beneficial for personalized surveillance protocols and treatment strategies.

## Materials and methods

The present retrospective study on CT enhancement of PDAC patients to predict TTR after upfront surgery was approved by the ethical committee of Heidelberg University, Germany (S-711/2021). The experimental protocol (including the retrospective search for patients in the local radiological database, pseudonymized export of CT files, placement of ROIs in CTs, and correlation of CT attenuation/enhancement values with prognostic and histopathological parameters) was approved by the ethical committee of Heidelberg University. Informed consent was waived in accordance with the data protection law of Baden-Württemberg § 13 by the ethical committee of Heidelberg University since obtainment of informed consent would have entailed a disproportionately high effort.

The present study is an exploratory study and not a confirmatory study of previously published results since most previous studies on CT enhancement quantification of PDAC used different endpoints (Supplementary Table 1).

### Patients

Inclusion criteria were: (1) upfront surgery with (partial) pancreatectomy without neoadjuvant chemo- and/or radiotherapy between 01/01/2011 and 01/01/2022, (2) final histopathological diagnosis of PDAC, and (3) available preoperative three-phase abdominal CT-examination (unenhanced, pancreatic parenchymal/late arterial phase and portal venous phase).

Exclusion criteria were: (1) inadequate image quality, (2) tumor extension in CT not clear ((partially) occult tumors or severe concomitant pancreatitis), and (3) recurrence-free patients with follow-up after surgery < 24 months.

The CT scanning protocols varied (see Supplementary Materials page 21).

The following biochemical and clinical parameters were collected and analyzed for all patients: age, sex, histopathological diagnosis, radiological tumor size, TNM stage (8th edition), histopathological grading, UICC stage, serum levels of tumor markers CA19-9, and CEA, pancreas resection (date, type), date of recurrence, type of recurrence, date of last follow-up.

TTR was defined as the time from surgery to detection of locoregional recurrence and/or distant metastases on follow-up CT scans. The imaging definition of tumor recurrence is described in the Supplementary Materials (page 3).

### Image analysis of the preoperative CT scan

Image analyses were performed by two board-certified radiologists in consensus, who were blinded to the patients’ outcomes, using a free and open-source code software (Horos (LGPL-3.0)). After coregistration of the axial images of the triple-phasic CT examination, the pancreatic tumor was identified, the slice with the maximal tumor dimension was selected, and ROIs were placed in the tumor, non-neoplastic parenchyma, and the aorta (Fig. 1 and Supplementary Materials (page 3)). ROIs were copied between each phase. Hounsfield Units (HU) were extracted from each ROI in each phase.

### Enhancement studies/formulas

The PubMed^®^ MEDLINE and PubMed Central^®^ search for identification of CT enhancement studies is described in the Supplementary Materials (page 4).

### Statistical analysis

Statistical analyses were conducted with MedCalc (Version 22.016, MedCalc Software Ltd., Ostend, Belgium) and R software (Version 4.3.2, https://www.r-project.org) using the compareC package (Version 1.3.2, https://CRAN.r-project.org/package=compareC). Descriptive analysis of variables is presented as median with IQR.

Power size calculation for TTR analysis was conducted. Previously reported Hazard Ratios (HRs) of CT enhancement measurements for prediction of tumur recurrence ranged between 1.96 and 7.1 and were used as a basis for the analysis^13,16,31^. Assuming HR = 1.96, α_two-tailed_ = 0.05, β = 0.2 (power = 0.80), an even distribution of groups (median as cutoff value), a baseline event rate of 0.59 (events/year = 1-year recurrence rate from Li et al.^41^), and a follow-up until recurrence or for ≥ 2 years, a sample size of 87 patients is required.

Mann–Whitney-U test and Wilcoxon Test were used to compare continuous variables between independent and paired samples. ANOVA with one-way analysis of variance was used to test differences between means of subgroups of a variables. Chi-squared Test was used to compare categorical variables between groups. Spearman’s correlation coefficient was obtained to define the correlation between continuous variables. TTR analysis was performed using Kaplan–Meier analysis and log-rank test as well as Cox proportional hazard regression analysis. In univariate Cox regression analysis, the ENTER method was used. For multivariate Cox regression analysis, variables were entered sequentially (FORWARD method) if their associated significance level was < 0.05 and variables were removed if their associated significance level was > 0.10, or all variables were entered in the model in one single step (ENTER method). Harrell’s C-indices were computed and compared for Cox models. ROC curve analysis was performed to evaluate the discriminatory ability of continuous and ordinal variables for the correct assignment of cases into cases without and with tumor recurrence at 1 year after surgery. Standard errors of AUC values and differences between two AUC values were calculated using the DeLong method.

In the present exploratory study, no alpha adjustment for multiple testing was done, as suggested by Bender & Lange^48^ and Althouse^49^. Alpha adjustment would increase the rate of false-negative findings (type II error), meaning that true correlations between enhancement values and clinical variables could be missed. However, using this approach, there is an increased chance of false-positive findings. Therefore, the positive findings (p < 0.05) from the present study should be tested in future confirmatory studies^48^.

### Supplementary Information

Supplementary Information.

## Data Availability

The datasets generated during and/or analyzed during the current study are available from the corresponding authors upon reasonable request.

## Abbreviations

AI: Artificial intelligence
AJCC: American Joint Committee of Cancer
ANOVA: Analysis of variance
α-SMA: Alpha-smooth muscle actin
art: Arterial
AUC: Area under the Curve
CA19-9: Carbohydrate antigen 19-9
CEA: Carcinoembryonic antigen
CT: Computed tomography
DFS: Disease-free survival
HR: Hazard Ratio
HU: Hounsfield units
IQR: Interquartile range
KRAS: Kirsten rat sarcoma virus
MDCT: Multidetector computed tomography
MVD: Microvessel density
NAT: Neoadjuvant therapy
nc: Non-contrast
OS: Overall survival
PDAC: Pancreatic cancer
RFS: Recurrence-free survival
ROC: Receiver operating characteristic
ROI: Region of interest
r: Spearman rank correlation coefficient
TNM: Tumor-Node-Metastasis
TP53: Tumor protein 53
TTR: Time to recurrence
Tu: Tumor
UICC: Union for International Cancer Control
Upstream: Upstream parenchyma
VEGF: vascular endothelial growth factor
ven: Portal venous
