# Supplementary material for: Performance of different CT enhancement quantification methods as predictors of pancreatic cancer recurrence after upfront surgery
Source: Sci Rep. 2024 Aug 26;14:19783. doi: 10.1038/s41598-024-70441-3 (PMC11347575; doi:10.1038/s41598-024-70441-3)
Supplement: Supplementary file 1 — Supplementary Information. [file 41598_2024_70441_MOESM1_ESM.pdf]

Supplementary material.

Performance of different CT enhancement quantification methods as predictors of pancreatic cancer recurrence after upfront surgery.

Sherif A Mohamed [1,2 +*], Alina Barlemann [1+], Verena Steinle [1], Tobias Nonnenmacher [1], Michelle Güttlein [1], Thilo Hackert [3], Martin Loos [4], Matthias M Gaida [5,6], Hans-Ulrich Kauczor [1], Miriam Klauss [1], Philipp Mayer [1*].

1 Clinic for Diagnostic and Interventional Radiology, Heidelberg University Hospital, Heidelberg, Germany.

2 Department of Neuroradiology, Medical Faculty Mannheim, Heidelberg University, Mannheim, Germany.

3 Department of General, Visceral and Thoracic Surgery, University Hospital Hamburg-Eppendorf, Hamburg, Germany.

4 Clinic of General, Visceral, and Transplantation Surgery, Heidelberg University Hospital, Heidelberg, Germany.

5 Institute of Pathology, University Medical Center Mainz, JGU-Mainz, Mainz, Germany

6 TRON, Translational Oncology at the University Medical Center, JGU-Mainz, Mainz, Germany.

+ Sherif A Mohamed and Alina Barlemann share first authorship and have provided equal contribution.

* Corresponding authors.

Supplementary Figure 1. Flowchart of the study population. 189 consecutive PDAC patients who underwent upfront surgery and had a preoperative three-phase abdominal CT examination were retrospectively identified from our radiological database. Excluded were 2 patients with inadequate CT image quality, 22 patients where the exact tumor extension in CT was not clear ((partially) occult tumor and/or severe concomitant pancreatitis), and 73 recurrence-free patients with follow-up <2 years. The final study population consisted of 92 PDAC patients with a median age of 67 years (interquartile range (IQR) 59 - 74 years).

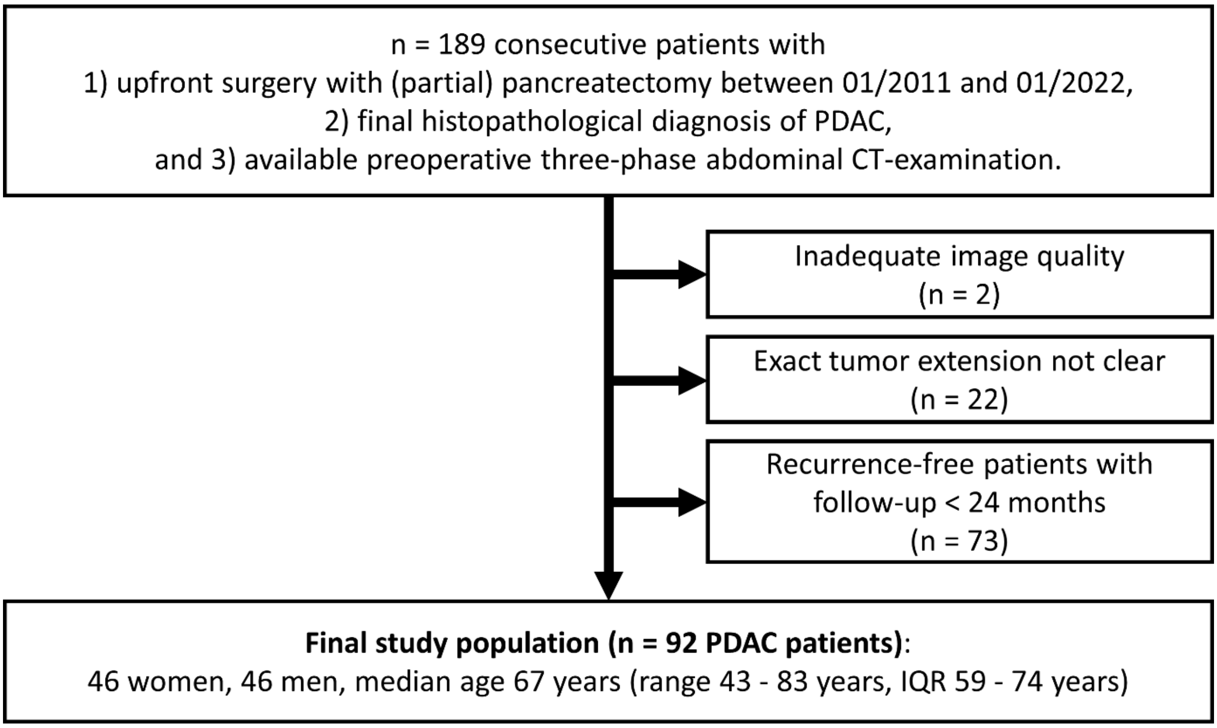

Pancreatic resections:

The following types of pancreas resection were performed: 29 patients received pylorus-preserving pancreaticoduodenectomy, 32 patients received conventional pancreaticoduodenectomy, 20 patients received left-sided pancreatic resection, and 11 patients underwent total pancreatectomy. Among these, three patients had concomitant distant metastasis: one patient with solitary liver metastasis had received simultaneous atypical liver segment resection, one patient with a distant lymph node metastasis an extended lymphadenectomy, and one patient with a solitary perisplenic peritoneal metastasis an extended multivisceral resection.

Imaging definition of tumor recurrence:

According to Kovač et al. [50] locoregional recurrence was defined as detection of new solid soft tissue or unequivocal progression of preexisting solid soft tissue in the resection area (often adjacent to major vessels) in comparison to the first postoperative CT scan (excluding early postoperative CT scans with extensive inflammatory changes in the resection area). Lymph node metastases were defined as increase in short axis diameter by > 50% AND > 1cm total short axis diameter, compared to the first postoperative CT scan. According to Holzapfel et al. [51], liver metastases were defined as new (mostly hypovascular) mass-like liver lesions not showing the typical imaging features of benign lesions (cysts, hemangiomas, focal nodular hyperplasia, focal fatty infiltration). Peritoneal recurrence was defined as occurrence of new peritoneal nodules > 5mm and/or peritoneal cake. New ascites was not considered sufficient for diagnosing peritoneal recurrence [52]. Lung metastases were defined as new or unambiguously progressive pulmonary nodules that are not typical for inflammatory changes [53]. Bone metastases were defined as new osteolytic or osteoblastic lesions that cannot be explained by degenerative changes or infection [54]. In equivocal cases, lesions suspicious of metastases had to be confirmed by subsequent follow-up scans; these scenarios include: 1) Difficult differential diagnosis of locoregional lymph node recurrence with the frequently occurring postoperative reactive lymphadenopathies (which usually regress within 6 months) [55]; 2) differential diagnosis of liver metastases with hepatic abscesses in patients with a biliodigestive anastomosis and clinical and/or laboratory signs of infection/cholangitis; 3) differential diagnosis of peritoneal recurrence with fat necrosis after surgery or after postoperative pancreatitis [52]; 4) equivocal lung lesions (e.g. lesions with predominantly alveolar presentation), especially in the presence of signs of infection [53]; 5) equivocal bone lesions which could possibly represent degenerative or infectious changes [54].

Placement of regions of interest (ROIs) in the preoperative CT scans:

The following ROIs were placed in every patient: 1) tumor: oval/ round, centered in the middle of the tumor, with a margin of at least 2 mm to the border of the tumor, avoiding calcifications and non-neoplastic tissue, 2) tumor periphery: freehand, approximately 5mm thick, peripheral tumor part [31], 3) aorta: oval/ round, in the aortic lumen, diameter ~15mm, avoiding plaques.

Auxiliary CT findings (location of duct cut-off, length of duct stenosis, loss of normal lobulation, contour deformity etc.) and -if available- information from preoperative magnetic resonance imaging scans were used to improve the anatomical outline of the tumor.

The following ROIs were placed in non-neoplastic pancreatic tissue (avoiding the pancreatic/ bile ducts and calcifications): 4) downstream parenchyma (towards the papilla): oval/ round, 5) upstream parenchyma (towards the tip to the pancreatic tail) - best measurable: oval or freehand, best measurable area of upstream non-neoplastic tissue, 6) upstream parenchyma - border to tumor: freehand, upstream parenchyma directly adjacent to the tumor. The ROIs in the non-neoplastic pancreatic tissue were only placed when possible: Upstream ROIs were not placed in cases with severely atrophic upstream parenchyma when valid attenuation measurements without partial volume effects were not possible or in cases with tumors at the tip of the pancreatic tail. Downstream ROIs were not placed if a pancreatic head tumor involved the papillary region.

ROIs were copied between each phase. If necessary, minor adjustments of copied in form and/or position were made. Hounsfield Units (HU) were extracted from each ROI in each phase (Figure 1 in main document).

Identification of CT enhancement studies and extraction of enhancement formulas:

A PubMed® MEDLINE and PubMed Central® search was performed using the search term "("Pancreatic Neoplasms"[Mesh]) AND (computed tomography OR CT) AND (enhancement OR enhanced)" for the time interval from 01/01/2009 to 01/31/2024. Studies were identified that used CT enhancement measurements of PDAC to predict clinical oncological/ time-to-event endpoints (such as TTR, OS…) and/or predict prognostically relevant histopathological tumor features (such as tumor grading, cellularity, stroma content…). 1189 (PubMed® MEDLINE) and 3845 (PubMed Central®) abstracts and articles were screened by two board-certified radiologists in consensus. 16 suitable studies were identified; all of which were retrospective. Extracted enhancement formulas are summarized in Supplementary Table 1.

Supplementary Table 1. Overview of included enhancement measurements/ formulas and predicted variables from previous studies

Studies	Enhancement measurements/ formulas	Predicted variables
**Enhancement of tumor without normalization to non-tumor structures**		
Bai X et al. 2023 [15]	(1) Tu_{art}<br>(2) Tu_{ven}	- occult metastasis
Fukukura Y et al. 2014 [21]	(3) $Tu_{art} - Tu_{nc}$<br>(4) $Tu_{ven} - Tu_{nc}$	- OS<br>- tumor cellularity<br>- stroma content
Goto S et al. 2021 [23] (see footnotes)	In addition to formula (3) from Fukukura Y et al. 2014:<br>(5) $Tu_{ven} - Tu_{art}$	- MVD<br>- tumor cellularity<br>- cancer-associated fibroblasts
Cai X et al. 2020 [16]	In addition to formulas (3) and (4) from Fukukura Y et al. 2014:<br>(6) Tu_{art}/Tu_{nc}<br>(7) Tu_{ven}/Tu_{nc}	- OS<br>- DFS<br>- stroma content<br>- histopathological grade
Park J et al. 2019 [22]	(8) $Tu_{art} - Tu_{nc}/Tu_{nc}$	- OS<br>- RFS
Cheng CS et al. 2019 [56]	(9) $Tu_{ven} - Tu_{nc}/Tu_{nc}$	- OS<br>- tumor stage
**Enhancement of tumor normalized to aortic enhancement**		
Seo W et al. 2020 [57]	(10) $Tu_{art}/Aorta_{art}$<br>(11) $Tu_{ven}/Aorta_{ven}$<br>(12) $\frac{Tu_{ven}-Tu_{art}}{Aorta_{art}-Aorta_{ven}}$	- histopathological grade
Jungmann F et al. 2021 [14]	Formula (11) from Seo W et al. 2020.	- tumor cellularity
Torphy RJ et al. 2018 [13]	(13) $\frac{Tu_{art}-Tu_{nc}}{Aorta_{art}-Aorta_{nc}}$<br>(14) $\frac{Tu_{ven}-Tu_{nc}}{Aorta_{ven}-Aorta_{nc}}$	- TTR after resection<br>- RFS<br>- OS<br>- stroma content
Hattori Y et al. 2009 [27]	Formulas (10) and (11) from Seo W et al. 2020 and formulas (3) and (4) from Fukukura Y et al. 2014.	- MVD<br>- VEGF expression<br>- fibrous stroma content

Supplementary Table 1 *continued*.

Studies	Enhancement measurements/ formulas	Predicted variables
**Enhancement of tumor in relation to non-neoplastic parenchyma**		
Fukukura Y et al. 2020 [6]	(15) $Upstream_{art} - Tu_{art}$<br>(16) $Upstream_{ven} - Tu_{ven}$	- OS<br>- PFS
Zhu L et al. 2016 [29]	In addition to formula (5) from Goto S et al. 2021:<br>(17) $\frac{Tu_{ven} - Tu_{art}}{Upstream_{ven} - Upstream_{art}}$	- OS<br>- PFS<br>- Expression of fibrogenic markers: α-SMA and perostin
Vyas SJ et al. 2016 [26]	In addition to measurement (1) from Bai X et al. 2023:<br>(18) ${Tu_{art}}/{Upstream_{art}}$	- OS<br>- histopathological grade
Gao JF et al. 2022 [17]	(19) $\frac{Tu_{ven} - Tu_{nc}}{Upstream_{ven} - Upstream_{nc}}$	- OS<br>- tumor stage<br>- tumor size<br>- lymph node metastases
**Enhancement of interface between tumor and non-neoplastic parenchyma**		
Koay EJ et al. 2018 [30]	(20) $Upstream_{border,art} - Tu_{periphery,art}$<br>(21) $Upstream_{border,ven} - Tu_{periphery,ven}$	- OS<br>- tumor stage<br>- distant metastasis-free survival<br>- response to neoadjuant therapy<br>- stroma content<br>- tumor cell phenotype<br>- KRAS/TP53 mutations
Amer AM et al. 2020 [31]		- OS<br>- time to distant metastasis<br>- lymphovascular invasion
Cai X et al. 2020 [16] (see also above under table section „Enhancement of tumor without normalization…“)	In addition to formulas (19) and (20) from Koay et al. 2018 and Amer AM et al. 2020:<br>(22) ${Upstream_{border,art}}/{Tu_{periphery,art}}$<br>(23) ${Upstream_{border,ven}}/{Tu_{periphery,ven}}$	- OS<br>- DFS<br>- stroma content<br>- histopathological grade

Formulas including attenuation measurements in the delayed phase were not included in the present study since delayed phase images were only available in a minority of patients and routine acquisition of a delayed phase is not recommended by current guidelines [10,11]. In the present study, the mean value of CT attenuation of each region of interest in each patient was used since mean HU values are less dependent on the specific reconstruction kernel than maximum attenuation values. Analyses of the non-neoplastic pancreatic parenchyma were performed on upstream parenchyma since downstream parenchyma was available in fewer patients.

Regarding the formulas from Goto et al. [23], differences in HU values were calculated instead of slopes since the exact time interval between arterial and portal venous phase was not known for every patient in our study.

Please note that enhancement values formula (8) are smaller by exactly 1 compared to values from formula (6): $Formula(8) = \frac{Tu_{art} - Tu_{nc}}{Tu_{nc}} = \frac{Tu_{art}}{Tu_{nc}} - \frac{Tu_{nc}}{Tu_{nc}} = \frac{Tu_{art}}{Tu_{nc}} - 1 = Formula(6) - 1$.

The same applies to formulas (9) and (7).

Abbreviations: art, late arterial; α-SMA, alpha-smooth muscle actin; DFS, disease-free survival; HU, Hounsfield units; KRAS, Kirsten rat sarcoma virus; MVD, microvessel density; nc, non-contrast; OS, overall survival; PFS, progression-free survival; RFS, recurrence-free survival; TP53, tumor protein 53; Tu, tumor; ven, portal venous; TTR, time to recurrence; Upstream, upstream parenchyma; VEGF, vascular endothelial growth factor.

Supplementary table 2. Median attenuation values (with interquartile ranges) in Hounsfield units

	n	Non-contrast phase	Late arterial phase	Portal venous phase
**Tumor**	92	32.2 (27.4-35.8)	58.6 (47.4-67.8)	70.7 (58.9-86.5)
**Tumor periphery**	92	32.0 (26.7-37.5)	59.8 (48.3-74.3)	74.1 (59.8-84.9)
**Upstream parenchyma - best measurable**	72	28.3 (20.4-35.3)	84.5 (66.6-106.5)	88.4 (76.1-100.5)
**Upstream parenchyma - border to tumor**	72	32.8 (27.7-38.8)	89.0 (63.2-113.0)	95.7 (78.2-109.1)
**Downstream parenchyma**	45	38.4 (32.5-44.1)	116.2 (90.6-129.4)	90.3 (82.0-98.9)
**Aorta**	92	43.0 (40.3-45.8)	297.2 (226.8-347.4)	138.3 (126.7-155.5)

Tumor attenuation was higher in the portal venous than in the late arterial phase ($p < 0.001$). Attenuation values of the tumors were lower than the attenuation values of the upstream (best measurable) and downstream parenchyma in the late arterial and portal venous phase ($p < 0.001$).

Downstream parenchyma showed an enhancement peak in the late arterial phase compared to the portal venous phase ($p < 0.001$). This enhancement peak was absent in upstream parenchyma.

The tumor-upstream interface attenuation was evaluated in 72 patients with measurable upstream parenchyma: CT attenuation values were lower in the tumor periphery than in the adjacent upstream parenchyma (border to the tumor), both in the late arterial and portal venous phase ($p < 0.001$).

Correlation of enhancement values from enhancement formulas with clinicopathological parameters:

Enhancement values from all formulas were not different in female versus male patients ($p \geq 0.0932$, $n = 72\text{-}92$).

Above median-age patients (≥ 67 years) had higher enhancement values than below median age patients (< 67 years) from formulas (2), (4), (7), (9), (11), (14) ($p \leq 0.034$, $n = 92$), as well as (17) ($p = 0.012$, $n = 72$). Above median-age patients also had lower values from formulas (20) and (22), corresponding to high peripheral tumor enhancement in relation to adjacent upstream parenchyma ($p \leq 0.029$, $n = 72$).

Arterial and portal venous attenuation values of the upstream and downstream parenchyma were not different between both age groups ($p \geq 0.5425$).

Low or intermediate-grade tumors ($G \leq 2$) had higher enhancement values than high-grade tumors ($G \geq 3$) from formulas (2), (10), (11), (14) ($p \leq 0.021$, $n = 92$).

Compared to patients with lymph-node metastasis(es), patients without lymph-node metastasis(es) had higher enhancement values from formulas (1), (3), and (13) ($p \leq 0.031$, $n = 92$). Lymph-node-negative patients also had lower values from formulas (5) and (12) ($p \leq 0.020$, $n = 92$) which quantitate the increase in tumor attenuation from late arterial to portal venous phase, without and with normalization to the aorta.

Compared to patients with UICC stage ≥ 3, patients with UICC stage ≤ 2 had higher enhancement values from formulas (1), (3), (6), (8) ($p \leq 0.042$, $n = 92$), but lower values from formula (12) which reflects the arterio-venous tumor attenuation difference (see above) ($p = 0.011$, $n = 92$).

Enhancement values from all formulas were not different between patients with $T \leq 2$ compared to patients with $T \geq 3$ ($p \geq 0.105$, $n = 72\text{-}92$).

There were weak negative rank correlations between radiological tumor size and enhancement values from formulas (3), (6), (7), (8), (9), and (13) ($-0.258 < r_s < -0.206$; $p \leq 0.048$, $n = 92$).

Supplementary Table 3. Results from the Kaplan-Meier analyses and log-rank test for different enhancement measurements/ formulas

Factor	n	Median TTR (95% CI) [days]	p-value (log-rank test)
Measurement (1) Tu_{art}			
≤ 58.6 HU	46	299 (218- 321)	**0.004**
> 58.6 HU	46	472 (305-684)	
Measurement (2) Tu_{ven}			
≤ 70.7 HU	46	291 (207-325)	**0.005**
>70.7 HU	46	399 (305-684)	
Formula (3) $Tu_{art} - Tu_{nc}$			
≤ 24.8 HU	46	299 (207-325)	**0.005**
> 24.8 HU	46	464 (305-684)	
Formula (4) $Tu_{ven} - Tu_{nc}$			
≤ 41.0 HU	46	306 (218-377)	**0.022**
> 41.0 HU	46	368 (294-682)	
Formula (5) $Tu_{ven} - Tu_{art}$			
≤ 12.4 HU	46	315 (232-527)	0.969
> 12.4 HU	46	318 (258-445)	
Formula (6) ${}^{Tu_{art}}/{}_{Tu_{nc}}$			
≤ 1.793	46	299 (207-338)	**0.027**
> 1.793	46	437 (305-627)	
Formula (7) ${}^{Tu_{ven}}/{}_{Tu_{nc}}$			
≤ 2.241	46	314 (218-437)	0.067
> 2.241	46	330 (264-544)	
Formula (8) ${}^{Tu_{art} - Tu_{nc}}/{}_{Tu_{nc}}$			
≤ 0.793	46	299 (207-338)	**0.027**
> 0.793	46	437 (305-627)	
Formula (9) ${}^{Tu_{ven} - Tu_{nc}}/{}_{Tu_{nc}}$			
≤ 1.241	46	314 (218-437)	0.067
> 1.241	46	330 (264-544)	
Formula (10) ${}^{Tu_{art}}/{}_{Aorta_{art}}$			
≤ 0.192	46	305 (230-338)	0.058
> 0.192	46	464 (294-606)	
Formula (11) ${}^{Tu_{ven}}/{}_{Aorta_{ven}}$			
≤ 0.528	46	306 (230-377)	0.215
> 0.528	46	368 (291-509)	
Formula (12) $\frac{Tu_{ven} - Tu_{art}}{Aorta_{art} - Aorta_{ven}}$			
≤ 0.086	46	311 (220-472)	0.714
> 0.086	46	338 (291-507)	
Formula (13) $\frac{Tu_{art} - Tu_{nc}}{Aorta_{art} - Aorta_{nc}}$			
≤ 0.108	46	291 (218-318)	**0.002**
> 0.108	46	509 (311-717)	

Supplementary Table 3 *continued.*

Factor	n	Median TTR (95% CI) [days]	p-value (log-rank test)
Formula (14) $\frac{Tu_{ven}-Tu_{nc}}{Aorta_{ven}-Aorta_{nc}}$			
≤ 0.440	46	309 (230-377)	0.130
> 0.440	46	368 (291-543)	
Formula (15) $Upstream_{art} - Tu_{art}$			
≤ 22.5 HU	36	305 (220-464)	0.426
> 22.5 HU	36	325 (264-602)	
Formula (16) $Upstream_{ven} - Tu_{ven}$			
≤ 15.4 HU	36	305 (220-544)	0.584
> 15.4 HU	36	318 (291-527)	
Formula (17) $\frac{Tu_{ven}-Tu_{art}}{Upstream_{ven}-Upstream_{art}}$			
≤ 0.486	36	325 (230-606)	0.328
> 0.486	36	314 (232-445)	
Formula (18) $Tu_{art}/Upstream_{art}$			
≤ 0.737	36	377 (306-602)	0.213
> 0.737	36	294 (171-399)	
Formula (19) $\frac{Tu_{ven}-Tu_{nc}}{Upstream_{ven}-Upstream_{nc}}$			
≤ 0.722	36	309 (170-527)	0.131
> 0.722	36	368 (264-544)	
Formula (20) $Upstream_{border,art} - Tu_{periphery,art}$			
≤ 25.3 HU	36	309 (221-399)	0.330
> 25.3 HU	36	340 (258-602)	
Formula (21) $Upstream_{border,ven} - Tu_{periphery,ven}$			
≤ 19.3 HU	36	311 (232-527)	0.996
> 19.3 HU	36	315 (220-543)	
Formula (22) $Upstream_{border,art}/Tu_{periphery,art}$			
≤ 1.383	36	318 (294-445)	0.714
> 1.383	36	306 (167-543)	
Formula (23) $Upstream_{border,ven}/Tu_{periphery,ven}$			
≤ 1.254	36	368 (232-549)	0.772
> 1.254	36	314 (220-437)	

Abbreviations: art, late arterial; HU, Hounsfield units; nc, non-contrast; ven, portal venous; Tu, tumor; TTR, time to recurrence; Upstream, upstream parenchyma.

Supplementary Table 4. Results from the Kaplan-Meier analyses and log-rank test for clinical and pathological variables.

Factor	n	Median TTR (95% CI) [days]	p-value (log-rank test)
CT tumor size			
< 28 mm	45	340 (232-684)	**0.026**
≥ 28 mm	47	314 (258-399)	
T stage			
≤ 2	74	340 (306-509)	0.068
≥ 3	18	232 (170-314)	
N status			
N0	51	464 (311-627)	**0.003**
N+	41	294 (183-338)	
Grading			
≤ 2	57	445 (318-544)	**0.006**
3	35	280 (171-311)	
CA19-9 [20]			
< 100 U/ml	43	507 (306-606)	**0.012**
≥ 100 U/ml	49	305 (207-325)	
CEA [42]			
< 2.5 ng/mL	67	318 (291-437)	0.194
≥ 2.5 ng/mL	25	340 (218-684)	

Abbreviations: CA19-9, carbohydrate antigen 19-9, CEA, carcinoembryonic antigen; N, nodal; T, tumor; TTR, time to recurrence.

Supplementary Table 5. Univariate Cox regression analyses: Clinicopathological parameters as predictors of tumor recurrence.

Covariate	n	Hazard ratio (95% CI)	p-value	Harrel's C-index (95% CI)
age > 67 years *versus* ≤ 67 years	92	0.906 (0.577-1.423)	0.668	0.532 (0.474-0.589)
sex male *versus* female		1.374 (0.873-2.161)	0.170	0.542 (0.484-0.599)
CT tumor size ≥ 28 mm *versus* < 28 mm		1.682 (1.059-2.674)	**0.028**	0.541 (0.482-0.600)
T ≥ 3 *versus* T ≤ 2		1.672 (0.957 to 2.921)	0.071	0.546 (0.505-0.587)
N+ *versus* N0		1.960 (1.246-3.085)	**0.004**	0.582 (0.528-0.636)
G = 3 *versus* G ≤ 2		1.898 (1.197-3.010)	**0.007**	0.586 (0.534-0.639)
CA19-9 ≥ 100 U/ml *versus* < 100 U/ml [21]		1.794 (1.132-2.843)	**0.013**	0.579 (0.523-0.634)
CEA ≥ 2.5 ng/mL *versus* < 2.5 ng/mL [49]		0.705 (0.415-1.198)	0.196	0.519 (0.465-0.573)

Abbreviations: CA19-9, carbohydrate antigen 19-9, CEA, carcinoembryonic antigen; G, grade; N, nodal; T, tumor.

Supplementary Table 6. Multivariate Cox regression analyses (FORWARD): prediction of tumor recurrence.

Covariate	n	Hazard ratio (95% CI)	p-value (covariate)	p-value (model)	Harrel's C-index (95% CI)
**Model with clinicopathological parameters as predictors**					
T ≥ 3 *versus* T ≤ 2	92	not included in model		**<0.001**	0.628 (0.577-0.680)
N+ *versus* N0		2.045 (1.296-3.226)	**0.002**		
G = 3 *versus* G ≤ 2		1.990 (1.251-3.166)	**0.004**		
CA19-9 ≥ 100 U/ml *versus* < 100 U/ml [21]		not included in model			
**Model with clinicopathological variables & enhancement values from formula (3) as predictors**					
T ≥ 3 *versus* T ≤ 2	92	not included in model		**<0.001**	0.651 (0.592-0.709)
N+ *versus* N0		1.661 (1.016-2.714)	**0.043**		
G = 3 *versus* G ≤ 2		2.212 (1.371-3.570)	**0.001**		
CA19-9 ≥ 100 U/ml *versus* < 100 U/ml [20]		not included in model			
Formula (3) > median versus ≤ median		0.570 (0.344-0.944)	**0.029**		
**Model with clinicopathological variables & enhancement values from formula (13) as predictors**					
T ≥ 3 *versus* T ≤ 2	92	not included in model		**<0.001**	0.657 (0.606-0.707)
N+ *versus* N0		1.850 (1.159-2.952)	**0.010**		
G = 3 *versus* G ≤ 2		1.802 (1.121-2.899)	**0.015**		
CA19-9 ≥ 100 U/ml *versus* < 100 U/ml [21]		not included in model			
Formula (13) > median *versus* ≤ median		0.606 (0.373-0.983)	**0.043**		

Abbreviations: CA19-9, carbohydrate antigen 19-9, G, grade; N, nodal; T, tumor.

Supplementary Table 7. Multivariate Cox regression analyses (ENTER): prediction of tumor recurrence.

Covariate	n	Hazard ratio (95% CI)	p-value (covariate)	p-value (model)	Harrel's C-index (95% CI)
**Model with clinicopathological parameters as predictors**					
T ≥ 3 *versus* T ≤ 2	92	1.448 (0.818- 2.560)	0.204	<0.001	0.668 (0.616-0.721)
N+ *versus* N0		1.732 (1.065-2.817)	**0.027**		
G = 3 *versus* G ≤ 2		1.897 (1.189-3.028)	**0.007**		
CA19-9 ≥ 100 U/ml *versus* < 100 U/ml [21]		1.445 (0.889-2.350)	0.138		
**Model with clinicopathological variables & enhancement values from formula (3) as predictors**					
T ≥ 3 *versus* T ≤ 2	92	1.247 (0.696-2.234)	0.458	<0.001	0.676 (0.618-0.735)
N+ *versus* N0		1.414 (0.846-2.362)	0.346		
G = 3 *versus* G ≤ 2		2.103 (1.301-3.400)	**0.002**		
CA19-9 ≥ 100 U/ml *versus* < 100 U/ml [21]		1.532 (0.943-2.488)	**0.085**		
Formula (3) > median versus ≤ median		0.563 (0.335-0.947)	**0.030**		
**Model with clinicopathological variables & enhancement values from formula (13) as predictors**					
T ≥ 3 *versus* T ≤ 2	92	1.244 (0.683-2.265)	0.476	<0.001	0.676 (0.622-0.729)
N+ *versus* N0		1.674 (1.029-2.722)	**0.038**		
G = 3 *versus* G ≤ 2		1.772 (1.100-2.854)	**0.019**		
CA19-9 ≥ 100 U/ml *versus* < 100 U/ml [21]		1.356 (0.832-2.210)	0.222		
Formula (13) > median *versus* ≤ median		0.671 (0.401-0.122)	0.128		

Abbreviations: CA19-9, carbohydrate antigen 19-9, G, grade; N, nodal; T, tumor.

Supplementary Table 8. Multivariate Cox regression analyses (FORWARD): preoperative models for prediction of tumor recurrence.

Covariate	n	Hazard ratio (95% CI)	p-value (covariate)	p-value (model)	Harrel's C-index (95% CI)
**Model with radiological tumor size, CA 19-9 & enhancement values from formula (1) as predictors**					
CT tumor size > median versus ≤ median	92	not included in model		0.002	0.615 (0.550-0.679)
CA19-9 ≥ 100 U/ml *versus* < 100 U/ml [21]		1.629 (1.022-2.596)	**0.040**		
Formula (1) > median versus ≤ median		0.562 (0.354-0.895)	**0.015**		
**Model with radiological tumor size, CA 19-9 & enhancement values from formula (2) as predictors**					
CT tumor size > median versus ≤ median	92	not included in model		**<0.001**	0.628 (0.565-0.691)
CA19-9 ≥ 100 U/ml *versus* < 100 U/ml [21]		1.732 (1.093-2.745)	**0.019**		
Formula (2) > median versus ≤ median		0.550 (0.348-0.871)	**0.011**		
**Model with radiological tumor size, CA 19-9 & enhancement values from formula (3) as predictors**					
CT tumor size > median versus ≤ median	92	not included in model		**0.001**	0.615 (0.552-0.678)
CA19-9 ≥ 100 U/ml *versus* < 100 U/ml [21]		1.756 (1.107-2.781)	**0.017**		
Formula (3) > median versus ≤ median		0.534 (0.338-0.844)	**0.007**		
**Model with radiological tumor size, CA 19-9 & enhancement values from formula (13) as predictors**					
CT tumor size > median versus ≤ median	92	1.644 (1.033-2.617)	**0.036**	**0.001**	0.616 (0.549-0.683)
CA19-9 ≥ 100 U/ml *versus* < 100 U/ml [21]		not included in model			
Formula (13) > median versus ≤ median		0.494 (0.311-0.785)	**0.003**		

Abbreviation: CA19-9, carbohydrate antigen 19-9.

Supplementary Table 9. Multivariate Cox regression analyses (ENTER): preoperative models for prediction of tumor recurrence.

Covariate	n	Hazard ratio (95% CI)	p-value (covariate)	p-value (model)	Harrel's C-index (95% CI)
**Model with radiological tumor size, CA 19-9 & enhancement values from formula (1) as predictors**					
CT tumor size > median versus ≤ median	92	1.577 (0.990-2.510)	0.055	**0.001**	0.628 (0.560-0.697)
CA19-9 ≥ 100 U/ml *versus* < 100 U/ml [21]		1.596 (1.0007-2.545)	**0.0496**		
Formula (1) > median versus ≤ median		0.580 (0.364-0.924)	**0.022**		
**Model with radiological tumor size, CA 19-9 & enhancement values from formula (2) as predictors**					
CT tumor size > median versus ≤ median	92	1.567 (0.980-2.504)	0.061	**<0.001**	0.628 (0.565-0.691)
CA19-9 ≥ 100 U/ml *versus* < 100 U/ml [21]		1.732 (0.109-2.745)	**0.019**		
Formula (2) > median versus ≤ median		0.550 (0.348-0.871)	**0.011**		
**Model with radiological tumor size, CA 19-9 & enhancement values from formula (3) as predictors**					
CT tumor size > median versus ≤ median	92	1.439 (0.893-2.321)	0.135	**0.001**	0.624 (0.557-0.691)
CA19-9 ≥ 100 U/ml *versus* < 100 U/ml [21]		1.725 (1.088-2.734)	**0.020**		
Formula (3) > median versus ≤ median		0.581 (0.363-0.931)	**0.024**		
**Model with radiological tumor size, CA 19-9 & enhancement values from formula (13) as predictors**					
CT tumor size > median versus ≤ median	92	1.616 (1.015-2.572)	**0.043**	**<0.001**	0.632 (0.562-0.702)
CA19-9 ≥ 100 U/ml *versus* < 100 U/ml [21]		1.516 (0.943-2.437)	0.086		
Formula (13) > median versus ≤ median		0.548 (0.340-0.883)	**0.014**		

Abbreviation: CA19-9, carbohydrate antigen 19-9.

Remarks on the selection of predictor variables and the Cox variable enter method:

Adjuvant therapies were not chosen as predictor variable since therapy regimens were highly heterogeneous, and in some patients who received adjuvant therapy outside our tertiary referral center, detailed information on therapy regimens were not available. For the preoperative models, the median radiological tumor size (28mm) (and not 20mm/40mm from TNM) was used as cutoff to generate similar group sizes.

For entering variables in the Cox model, different methods are available. The ENTER method enters all variables in the model in one step. The FORWARD method sequentially enters variables in the model (if their associated p-value is < 0.05) starting with the most significant variable. After each step, p-values are recalculated. A variable is removed from the model if its associated p-value is >0.10 [58]. The FORWARD method is less susceptible to collinearity, i.e. intercorrelations between predictor variables[59], and might be advantageous for clinical prediction modeling.

Supplementary Table 10. ROC curve analyses of different enhancement measurements, clinical and pathological parameters for the presence of tumor recurrence at 1 year.

	n	AUC (95% CI)	p-value	Youden index	Associated criterion
**Enhancement measurements/ formulas**					
Measurement (1) Tu_{art}	92	0.69 (0.58-0.78)	**<0.001**	0.384	≤ 59.36 HU
Measurement (2) Tu_{ven}	92	0.64 (0.54-0.74)	**0.014**	0.300	≤ 64.83 HU
Formula (3) $Tu_{art} - Tu_{nc}$	92	0.67 (0.56-0.77)	**0.003**	0.339	≤ 19.96 HU
Formula (4) $Tu_{ven} - Tu_{nc}$	92	0.63 (0.53-0.73)	**0.023**	0.275	≤ 27.32 HU
Formula (5) $Tu_{ven} - Tu_{art}$	92	0.51 (0.40-0.62)	0.880	0.098	> -10.73 HU
Formula (6) ${Tu_{art}}/{Tu_{nc}}$	92	0.66 (0.55-0.75)	**0.007**	0.253	≤ 2.192
Formula (7) ${Tu_{ven}}/{Tu_{nc}}$	92	0.61 (0.51-0.71)	0.056	0.232	≤ 1.972
Formula (8) ${Tu_{art} - Tu_{nc}}/{Tu_{nc}}$	92	0.66 (0.55-0.75)	**0.007**	0.253	≤ 1.192
Formula (9) ${Tu_{ven} - Tu_{nc}}/{Tu_{nc}}$	92	0.61 (0.51-0.71)	0.056	0.232	≤ 0.972
Formula (10) ${Tu_{art}}/{Aorta_{art}}$	92	0.69 (0.58-0.78)	**0.001**	0.339	≤ 0.175
Formula (11) ${Tu_{ven}}/{Aorta_{ven}}$	92	0.65 (0.54-0.75)	**0.008**	0.262	≤ 0.564
Formula (12) $\frac{Tu_{ven}-Tu_{art}}{Aorta_{art}-Aorta_{ven}}$	92	0.58 (0.47-0.68)	0.202	0.182	≤ 0.031
Formula (13) $\frac{Tu_{art}-Tu_{nc}}{Aorta_{art}-Aorta_{nc}}$	92	0.70 (0.60-0.80)	**<0.001**	0.369	≤ 0.089
Formula (14) $\frac{Tu_{ven}-Tu_{nc}}{Aorta_{ven}-Aorta_{nc}}$	92	0.65 (0.55-0.75)	**0.007**	0.275	≤ 0.232
Formula (15) $Upstream_{art} - Tu_{art}$	72	0.52 (0.40-0.64)	0.784	0.205	> 50.37 HU
Formula (16) $Upstream_{ven} - Tu_{ven}$	72	0.59 (0.47-0.71)	0.167	0.240	> 19.80 HU
Formula (17) $\frac{Tu_{ven}-Tu_{art}}{Upstream_{ven}-Upstream_{art}}$	72	0.51 (0.39-0.63)	0.847	0.162	≤ 0.955
Formula (18) ${Tu_{art}}/{Upstream_{art}}$	72	0.54 (0.41-0.65)	0.611	0.231	≤ 0.495
Formula (19) $\frac{Tu_{ven}-Tu_{nc}}{Upstream_{ven}-Upstream_{nc}}$	72	0.62 (0.50-0.73)	0.067	0.249	≤ 0.841
Formula (20) $Upstream_{border,art} - Tu_{periphery,art}$	72	0.50 (0.38-0.62)	0.987	0.096	≤ 5.7 HU
Formula (21) $Upstream_{border,ven} - Tu_{periphery,ven}$	72	0.51 (0.39-0.63)	0.836	0.138	> 10 HU
Formula (22) ${Upstream_{border,art}}/{Tu_{periphery,art}}$	72	0.54 (0.42-0.66)	0.534	0.121	> 1.300
Formula (23) ${Upstream_{border,ven}}/{Tu_{periphery,ven}}$	72	0.56 (0.44-0.67)	0.408	0.224	> 1.361

Supplementary Table 10 *continued.*

	n	AUC (95% CI)	p-value	Youden index	Associated criterion
**Clinicopathological parameters**					
Tumor size	92	0.58 (0.47-0.68)	0.176	0.206	> 42 mm
T status		0.60 (0.49-0.70)	0.052	0.177	> 2
N status		0.63 (0.53-0.73)	**0.016**	0.233	N+
Grading		0.67 (0.57-0.77)	**<0.001**	0.334	> 2
CA19-9		0.64 (0.54-0.74)	**0.013**	0.291	> 134.9 U/mL
CEA		0.51 (0.40-0.61)	0.882	0.128	> 5.1 ng/mL

Abbreviations: art, arterial; AUC, area under curve; CA19-9, carbohydrate antigen 19-9, CEA, carcinoembryonic antigen; G, grade; HU, Hounsfield units; N, nodal; nc, non-contrast; ROC, receiver operating characteristic; T, tumor; Tu, tumor; Upstream, upstream parenchyma; ven, portal venous.

Supplementary Figure 2. Comparison of ROC curves for prediction of tumor recurrence at 1 year after surgery. Shown are the receiver operating characteristic (ROC) curves for the four enhancement measurements/ formulas with the highest Area under the Curve (AUC) values (0.67 ≤ AUC ≤ 0.70). All of these formulas analyzed the tumor (Tu) attenuation in the arterial (art) phase, some subtracted by the non-contrast (nc) attenuation, and some normalized to the aorta. For comparison, ROC curves for the two clinicopathological variables (histopathological grading and nodal (N) status) with the highest AUC values (0.63 ≤ AUC ≤ 0.67) are shown. Differences in the AUC of the shown ROC curves yielded p-values ≥ 0.288.

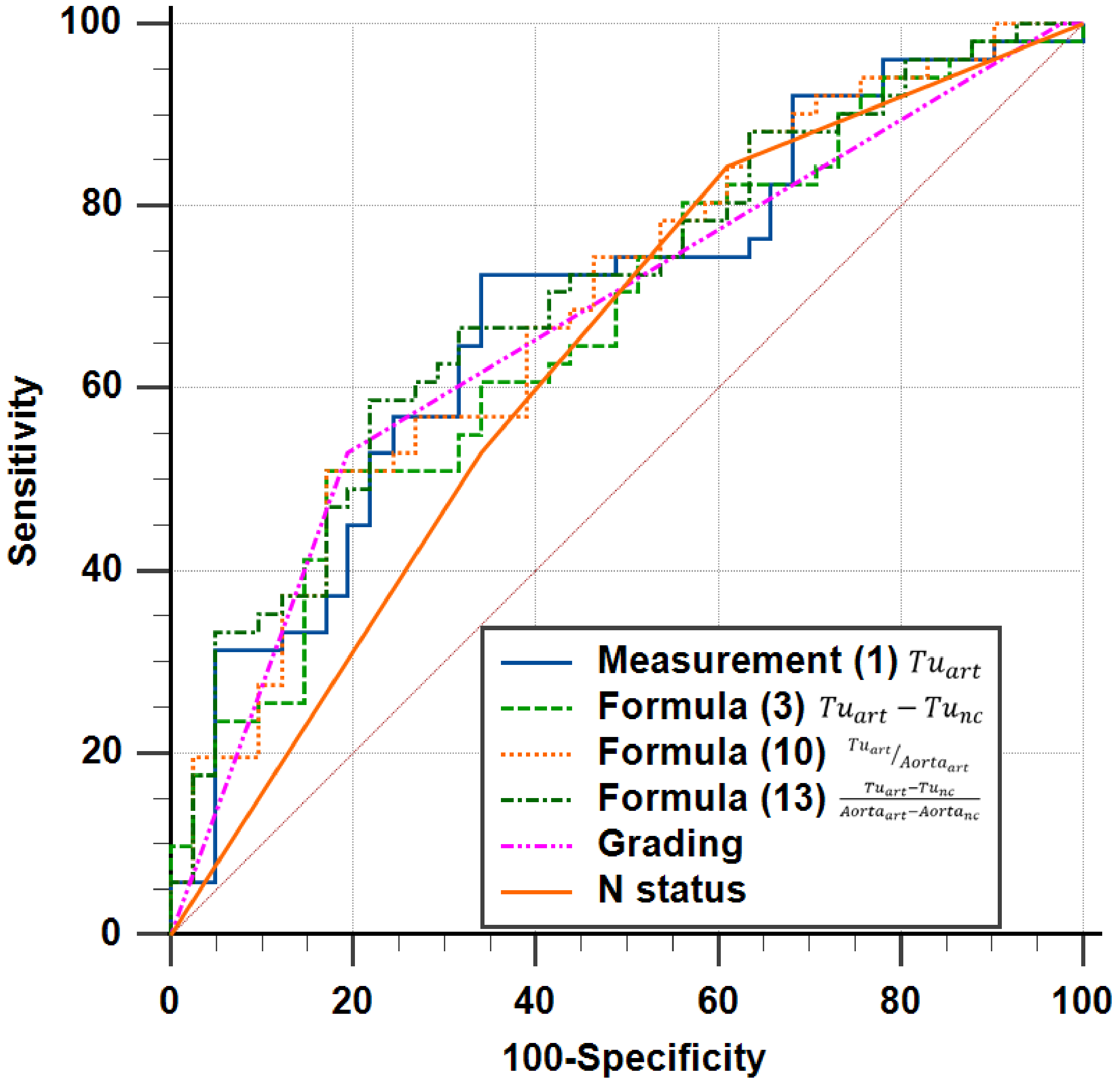

Technical parameters of CT scanning, their correlation with enhancement values, and their predictive value on time to recurrence:

Preoperative CTs were performed on scanners from Siemens Healthineers (Erlangen, Germany) with 6 to 384 rows (median 256 rows) in 75 patients, from Philips Healthcare (Amsterdam Netherlands) with 6 to 256 rows (median 256 rows) in 14 patients, and from Toshiba/Canon Medical Systems Europe (Amstelveen, Netherlands) with 16 to 80 rows (median 16 rows) in 3 patients. The images were reconstructed using filtered backprojection or iterative body kernels (42 and 50 patients). The reconstructed images had a median slice thickness of 1.5mm (interquartile range [IQR] 1 - 3 mm) and a median pixel spacing of 0.737 mm (IQR 0.689 - 0.807 mm). Reliable information on contrast agent volume (median 80ml, IQR 80 - 100 ml) and injection rate (median 5ml/s, IQR 3 - ml/s) were only available for 9 patients. Iodine concentration of contrast agent was available for 45 patients (median 350 mg/ml, IQR 350 - 350 mg/ml).

CT rows, slices thickness, pixel spacing, and iodine concentration as well as the distribution of CT manufacturers, CT models, and reconstruction kernels were not different between patients with or without recurrence at 1 year ($p \geq 0.084$) or 2 years ($p \geq 0.216$) after surgery. Image slice thickness was weakly positively rank-correlated with the enhancement values from formula (10) ($r_s = 0.342$, $p < 0.001$) and weakly negatively rank-correlated with the enhancement values from formula (15) ($r_s = -0.242$, $p = 0.040$). The values from all other enhancement measurements/ formulas were not correlated with image slice thickness ($p \geq 0.086$). The values from all enhancement formulas were not correlated with pixel spacing ($p \geq 0.063$) or iodine concentration ($p \geq 0.095$). Means of values from enhancement formulas were not different between scanner types ($p \geq 0.094$). Kaplan-Meier analyses with the log-rank test revealed no differences in TTR between preoperative CTs from Siemens versus non-Siemens models ($p = 0.218$), with filtered backprojection versus iterative reconstruction kernels ($p = 0.696$), ≤ versus > median slice thickness ($p = 0.823$), ≤ versus > median pixel spacing ($p = 0.643$), ≤ versus > median iodine concentration ($p = 0.135$). The combined clinical-radiological Cox proportional-hazards regression models (see main manuscript), which in addition to the clinical variables, included one of the four enhancement formulas/ measurements (1), (2), (3), (13), were recalculated (using the FORWARD and ENTER method) with one of the following CT scanning/ reconstruction parameters as additional predictor variable, each: CT manufacturer (Siemens versus non-Siemens), reconstruction kernel (filtered backprojection versus iterative), iodine concentration (≤ versus > median), slice thickness (≤ versus > median) and pixel spacing (≤ versus > median). Using the FORWARD method, the scanning/ reconstruction parameters were found not to contribute to the prediction of TTR and were not included in the final models. Using the ENTER method, CT manufacturer was associated with $p \geq 0.342$, reconstruction kernel with $p \geq 0.474$, iodine concentration with $p \geq 0.149$, slice thickness with $p \geq 0.580$, and pixel spacing with $p \geq 0.266$.

Additional references:

50. Kovač, J. D., Mayer, P., Hackert, T. & Klauss, M. The Time to and Type of Pancreatic Cancer Recurrence after Surgical Resection: Is Prediction Possible? *Acad Radiol* **26**, 775–781 (2019).
51. Holzapfel, K. et al. Comparison of diffusion-weighted MR imaging and multidetector-row CT in the detection of liver metastases in patients operated for pancreatic cancer. *Abdom Imaging* **36**, 179–184 (2011).
52. Chu, L. C. et al. Postoperative surveillance of pancreatic ductal adenocarcinoma (PDAC) recurrence: practice pattern on standardized imaging and reporting from the society of abdominal radiology disease focus panel on PDAC. *Abdom Radiol.* **48**, 318–339 (2022).
53. Aissaoui, M. et al. CT features of lung metastases from pancreatic adenocarcinoma: Correlation with histopathologic findings. *Diagn Interv Imaging* **102**, 371–377 (2021).
54. Isaac, A., Dalili, D., Dalili, D. & Weber, M.-A. State-of-the-art imaging for diagnosis of metastatic bone disease. *Radiologe* 60, 1–16 (2020).
55. Chincarini, M., Zamboni, G. A. & Pozzi Mucelli, R. Major pancreatic resections: normal postoperative findings and complications. Insights Imaging **9**, 173–187 (2018).
56. Cheng, C. et al. Prognostic Predicting Role of Contrast-Enhanced Computed Tomography for Locally Advanced Pancreatic Adenocarcinoma. *BioMed Res Int.* **2019**, 1–9 (2019).
57. Seo, W., Kim, Y. C., Min, S. J. & Lee, S. M. Enhancement parameters of contrast-enhanced computed tomography for pancreatic ductal adenocarcinoma: Correlation with pathologic grading. *World J Gastroenterol.* **26**, 4151–4158 (2020).
58. MedCalc- Manual. Statistics, Survival analysis, Cox proportional-hazards regression. https://www.medcalc.org/manual/cox-regression.php [accessed 10/07/2024].
59. Chowdhury, M. Z. I. & Turin, T. C. Variable selection strategies and its importance in clinical prediction modelling. *BMJ Fam Med Community Health* **8**, e000262 (2020).

For references 1 - 49 see the main document.
